# A262 THE EXCESS USE OF PROTON-PUMP INHIBITORS IN CHILDREN WITH INFLAMMATORY BOWEL DISEASE

**DOI:** 10.1093/jcag/gwad061.262

**Published:** 2024-02-14

**Authors:** N Singh, Z Nugent, W El-Matary, H Singh, S Shaffer, C Bernstein

**Affiliations:** University of Manitoba Max Rady College of Medicine, Winnipeg, MB, Canada; University of Manitoba Max Rady College of Medicine, Winnipeg, MB, Canada; University of Manitoba Max Rady College of Medicine, Winnipeg, MB, Canada; University of Manitoba Max Rady College of Medicine, Winnipeg, MB, Canada; University of Manitoba Max Rady College of Medicine, Winnipeg, MB, Canada; University of Manitoba Max Rady College of Medicine, Winnipeg, MB, Canada

## Abstract

**Background:**

Proton-pump inhibitors (PPI) use can impact the gut microbiome, thus, it is possible that use in children may be associated with an increase in pediatric IBD.

**Aims:**

We investigated the common gastrointestinal symptoms that result in a PPI prescription in pediatrics prior to a diagnosis of IBD and the degree to which children with IBD use excess PPIs compared to controls either prior to or post a diagnosis of IBD.

**Methods:**

The University of Manitoba IBD Epidemiology Database includes all Manitobans diagnosed with IBD 1984-2018 with age, sex, and geographic-matched controls. PPI prescription data were assessed from April 1995 onwards in children diagnosed with IBD prior to age 18.

**Results:**

PPI were dispensed prior to IBD diagnosis in 9% of 614 children diagnosed with IBD and 0.8% of 5718 controls (P ampersand:003C0.0001) with the median age being 15 years. Children with Crohn’s disease were no more likely to have been PPI-users pre-diagnosis (10%) than persons with ulcerative colitis (8%, p=0.57). Relative PPI use increases within 1-year of an IBD diagnosis (rate ratio, RR=22.4 (95% CI 13.6-37); P ampersand:003C 0.001) compared with 3-5 years pre-IBD (RR=4.9, 95% CI 1.4-16.7) and 1-3-year pre-IBD (RR=2.5, 95% CI 1.01-6.2). The percent of children being prescribed a PPI increased the more they visited a physician, which was associated with increased likelihood of being prescribed a PPI. PPI use was similar for the various gastrointestinal diagnoses in cases of IBD and controls. PPI users prior to a diagnosis may have less severe disease than non-PPI users. We found they were less likely to be hospitalized (HR 0.36, 95% CI 0.19 – 0.68; P = 0.002). There was no difference among children who had surgery for their IBD whether they were PPI users prior to IBD diagnosis compared to non-users.

**Conclusions:**

PPI use was commonly prescribed for a variety of gastrointestinal complaints, similarly in cases and controls. Children with IBD have many more contacts for gastrointestinal diagnoses than controls even for 5 years prior to their diagnosis. This raises the possibility that PPIs are prescribed indiscriminately for gastrointestinal complaints since their use was not restricted to complaints warranting PPI use, or that PPIs are prescribed for early symptoms that are secondary to IBD.

**Funding Agencies:**

None

